# A Realist Evaluation of a 72-Hour Readmission Audit and Feedback (A&F) Intervention in Emergency Medicine

**DOI:** 10.7759/cureus.18402

**Published:** 2021-09-30

**Authors:** William Kennedy, Shawn Dowling, Kevin Lonergan, Tom Rich, Catherine Patocka

**Affiliations:** 1 Internal Medicine, University of Saskatchewan, Regina, CAN; 2 Emergency Medicine, University of Calgary, Calgary, CAN; 3 Analytics, Data Integration, Measurement and Reporting Program, Alberta Health Services, Calgary, CAN

**Keywords:** qualitative research, feedback, realist evaluation, audit and feedback, emergency medicine

## Abstract

Introduction

Audit and feedback (A&F) interventions are intended to increase accountability and improve the quality of care; however, their impact can vary significantly. As performance feedback is implemented in healthcare, there is a growing need to determine how users interact with the data and how systems can achieve more consistent performance outcomes. This study aimed to understand the contexts, mechanisms, and outcomes of an emergency department 72-hour readmission A&F intervention.

Methods

Semi-structured interviews with key stakeholders were conducted and analyzed using thematic and template analysis techniques specifically aimed at identifying context, mechanism, and outcome configurations.

Results

Seventeen (17) physician interviews were conducted. We identified five outcomes of the intervention and the contexts and mechanisms contributing to them. Importantly, we identified that this A&F strategy could potentially have positive (improved follow-up of cases, improved discharge communication) and negative impacts (increased physician anxiety, potentially increased resource use) on physicians and departmental efficiency.

Conclusion

The 72-hour readmission alert A&F intervention generates a number of distinct outcome patterns that result from a variety of mechanisms acting in different contexts. Knowledge of these context-mechanism-outcome relationships may help implementers design and tailor performance feedback strategies.

## Introduction

Audit and feedback (A&F) is a quality improvement strategy that aims to improve care by providing clinicians individualized performance feedback [1]. Although A&F interventions are intended to increase accountability and improve quality of care [2], a Cochrane systematic review of A&F interventions suggested uncertainty in their effectiveness [1]. Existing studies of A&F in healthcare have not always considered the complex interactions between recipients, their context, and the components of the A&F intervention itself [3]. Given the growing desire for performance measures and feedback in medicine, it is important to understand how providers respond to different forms of A&F.

Seventy-two-hour readmission occurs when a patient is admitted to the hospital within 72 hours of discharge from a previous emergency department (ED) visit [4]. Although the 72-hour readmission window may be influenced by aspects beyond emergency care, it exists as one of the currently available measures to examine episodic care [4-5]. With the goal of promoting reflection and practice improvement, our ED implemented a 72-hour readmission A&F intervention. This study aimed to better understand how this intervention may influence clinician behaviour.

Realist evaluation, a theory-driven research approach, may be useful in advancing our understanding of process improvement efforts such as A&F [6-7]. This methodology uses context-mechanism-outcome (CMO) configurations to help highlight the behavioural or social aspects that influence complex interventions [7]. Context refers to pre-existing structures: organization norms, biases, and power structures that create regularities in the process [7]. Mechanisms are what make a process work or drive it and what about stakeholder choices or reasoning puts a process into action [7]. A mechanism is an individual’s personal account of how his or her behaviour and interaction with a process will ultimately influence it. Finally, outcomes are the result of mechanisms interacting within a context: the stakeholders reasoning within a particular context that results in intended or not unintended consequences [7].

As an example, we can apply the CMO configuration as follows: during a global pandemic (context), a person's belief that emergency departments are significant exposure sites for infection (mechanism), may result in a higher proportion of people willing to wear masks (outcome).

When applied to A&F interventions, realist evaluation offers an opportunity to go beyond examining an intervention’s intended effects and look at other outcomes that may be at play [8].

This study aimed to identify the potential outcomes of a 72-hour readmission A&F intervention and explain the mechanisms and contexts contributing to those outcomes in hopes of informing ongoing and future implementations of similar A&F interventions.

## Materials and methods

Realist evaluation

Realist philosophy suggests that programs work differently in discrete contexts because the mechanisms needed for success are triggered to varying degrees. To understand how the 72-hour readmission alert might generate distinct outcome patterns in various circumstances, this evaluation examined how mechanisms were triggered in particular contexts.

Intervention

Our institution is located in a large Canadian city with a population of ~1.4 million people. The 72-hour readmission alert was implemented using data from our local electronic health record, which allowed for an automated alert to be sent to individual emergency physicians notifying them of 72-hour readmission. The alert details included patient name, age/gender, facility, ED physician(s) involved in the patient’s care, presenting complaint, discharge time and diagnosis, re-admission date/time, admitting team, and admitting diagnosis.

Study team

Our study team consisted of a research assistant, a medical student, a data analyst, and three emergency physicians. The research assistant conducted all the interviews. The medical student had an interest in medical education, feedback, and leadership and felt that this project intersected well with those interests. The three emergency physicians had direct experience receiving 72-hour readmission alerts. The data analyst was directly involved in developing the alert. At the time of the study, two of the three emergency physicians held leadership/quality improvement roles within the department. We felt that the team was well-equipped to synthesize the data because we included relevant stakeholders (alert designers, implementers, recipients) as well as medical educators with an understanding of the formative intent of the feedback.

Prior to undertaking the realist evaluation, the study group met to develop an initial program theory (Appendix A). The initial program theory was formulated and summarized as a logic model because it was felt to be an appropriate way of representing the program and guiding the evaluation [9].

Data collection and analysis

Ethical approval for this study was obtained from the Conjoint Health Research Ethics Board (REB 18-1285), and participants provided informed verbal consent to participate in semi-structured interviews. All interviews were conducted by a trained research assistant between April and May 2019 in person or using an online platform. The interviewer followed an interview guide (Appendix B). Initially, we used purposeful random sampling of emergency physicians stratifying participants based on gender, time in clinical practice, and worksite. We subsequently used a targeted sampling of the leadership team to understand their rationale in implementing this A&F strategy. Sampling continued until we felt we had achieved theoretical sufficiency. Sufficiency was defined as no new outcome patterns emerging after two additional interviews.

Recorded interviews were transcribed by an experienced transcription service, and participants’ identities were redacted prior to analysis. Data were checked and errors were corrected by one of the authors (CP). All transcripts were independently reviewed and coded by two coders using a combination of thematic analysis, alongside context, mechanism, outcome (CMO) identification [7,10]. Once patterns of outcomes were identified, the mechanisms generating those outcomes were analyzed and the contexts in which particular mechanisms did or did not ‘fire’ were determined. The analytic process resulted in a set of CMO statements. Finally, the CMO configurations that offered the most robust and plausible explanation were determined and compared to the initial program theory to generate a revised program theory. Coding was conducted using NVivo (QSR International Pty Ltd., Victoria, Australia). We used a constant comparative method in that we built, discussed and amended theories, interpretations and explanations through the data collection and analysis stages [11]. The outputs for the analysis phase were a combination of codebooks, memos, and a tabulated list of CMO triads (Appendix C).

Synthesis

Drawing on the products of the analysis, the coders discussed and iteratively developed middle-range theories [12-13]. The study team met and reviewed the emerging themes identified by the coders to ensure they reflected the entire team’s understanding of the data. The logic model of the program theory developed was redrawn to reflect the findings. For clarity, the CMO configurations were subsequently organized into outcome patterns of interest and their contributing contexts and mechanisms.

## Results

Seventeen (17) participants (10 female, 7 male) with varying experience in clinical practice (10 months-30 years) were interviewed. All participants had received at least one 72-hour readmission alert and two held leadership positions. We identified five outcome patterns and contributory mechanisms and contexts. Outcome patterns were classified as having department- and physician-level impacts while mechanisms and contexts were classified as promoting or impeding a particular outcome pattern.

Department-level impacts of the alert

Outcome # 1: Perceived Increase in ED Resource Use

Participants felt that, in some instances, alert notifications caused them to increase (or consider increasing) their use of healthcare resources such as diagnostic imaging, consultation, and admission. This appeared to be driven by a number of mechanisms. Some participants had the perception that a 72-hour readmission alert was avoidable or reflected a mistake by the treating physician. Others expressed a desire to avoid 72-hour readmission alerts due to the stress they evoked. Finally, others outlined the perception that fewer 72-hour readmissions were desired by departmental leadership.

In contrast, some participants took the time to reflect and normalize 72-hour returns. They seemed to recognize that increasing resource use to avoid 72-hour readmissions would increase inefficiencies in the health system without improving patient outcomes.

Outcome # 2 Elaboration of Discharge Instructions

Respondents frequently chose to elaborate their discharge instructions (both verbal and written) in response to feedback they received from 72-hour readmission alerts. In most cases, physicians perceived the electronic discharge instructions as a means to communicate their rationale for discharging the patient home. This mechanism appeared to fire most frequently in the context of patient visits where a physician chose to discharge a patient home but felt there was a significant likelihood that the patient would return.

Instances where physicians were unlikely to elaborate discharge instructions included those where they felt readmission was unlikely or where the alert was considered inappropriate or unhelpful (patient returning for planned imaging or discharged by a consulting service).

**Figure 1:**
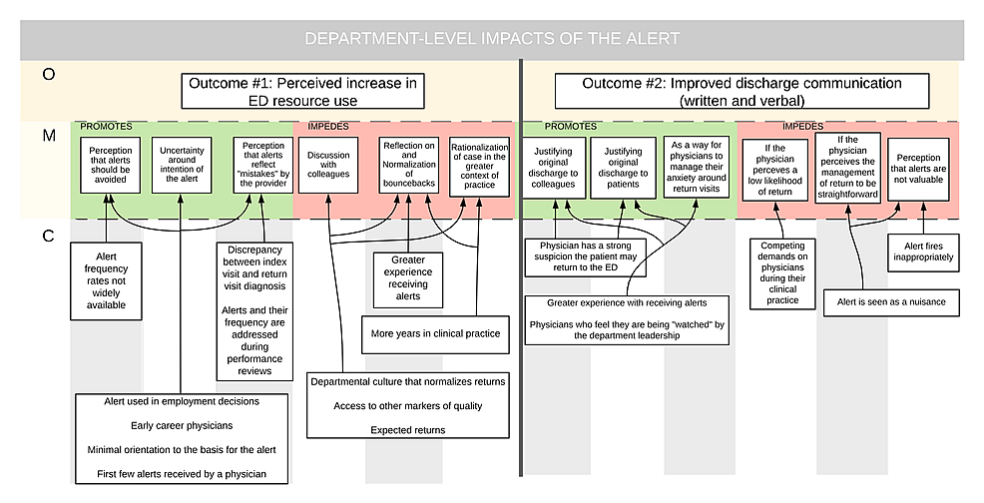
Department-level impacts of the alert

Figure 1 shows outcomes #1 and #2.

Individual physician-level impacts of the alert

Outcome #3: Increased Stress or Anxiety

Anxiety and stress were frequently perceived outcomes. There was variability in the degree of anxiety reported with some describing excessive stress and others describing a manageable amount of anxiety. Large amounts of stress were associated with the perception that the 72-hour readmission alert represented a mistake or failure in care, the perception that the alert reflected something that was avoidable through the actions of the individual physician, the belief that alert numbers and metrics were being tracked by the ED, and the perception that a patient returning as a ‘bounceback’ was different from a patient presenting with the natural progression of their symptoms. These particular mechanisms seemed to fire in a variety of contexts: for some, it was present in all instances where they received a trigger alert, for others, it was prominent in the presence of misfired trigger alerts. In particular, participants seemed to have heightened anxiety when their 72-hour readmission alert metrics had been discussed during their individual performance review. The mechanisms that seemed to lead to more manageable anxiety were the perception that the alert was not intended as punitive feedback and those who perceived information about disease progression and evolution as helpful to their learning. Finally, collegiality was a significant element of alert-associated stress, as those believing the institution to be a supportive environment were more likely to positively view alert feedback.

A context that was frequently associated with a heightened but manageable amount of anxiety was among physicians who had an established mechanism (other than trigger alerts) to follow-up their patients. These physicians held the belief that information about patient outcomes was helpful as it reinforced their practice and allowed them to learn from their previous experiences. They did, however, struggle with the alert “pushing” feedback automatically rather than their existing mechanisms of “seeking” feedback. Having received a number of anxiety-provoking alerts, physicians who believed they were not in an appropriate mindset to process the information developed strategies to delay reading the alert.

Outcome #4: Review of Individual Cases

Upon receiving a 72-hour readmission alert, participants frequently returned to the patient chart to review their own documentation as well as notes and results summarizing the return visit/admission. In many instances, the mechanism driving this process was curiosity and desire to understand the circumstances surrounding the return. This mechanism fired frequently in the context of unexpected return visits, where the physician was surprised to learn that the patient had returned. Another distinct but related context was when there was discordance between the diagnosis on discharge from the emergency department and readmission diagnosis within 72 hours.

**Figure 2:**
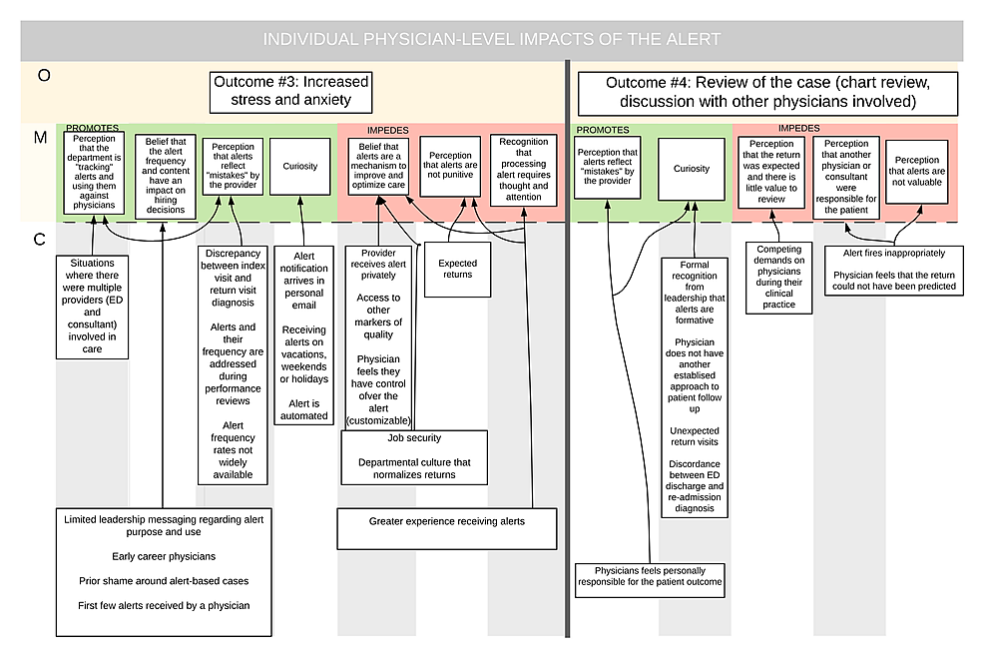
Individual physician-level impacts of the alert

Figure 2 shows outcomes #3 and #4.

Outcome #5: No Change in Behaviour

Physicians described many instances where they felt that the 72-hour readmission alert did not change their practice. In particular, for patients with expected or unsurprising visits, the physicians had the perception that the alert confirmed their practice and no change in behaviour or action was required. Some physicians found such instances of feedback helpful, as it provided important learning regarding the natural progression of illness and felt that it gave them confidence in their practice. Others found these instances of feedback a nuisance and would have preferred not to receive them as trigger alerts. Specifically, in the context of an expected return visit, the combined belief that the alert confirmed their practice and the perception that alerts were intended to identify mistakes or failure in care was problematic. These mechanisms together led to an outcome pattern, including no change in clinical behaviour, but the presence of a great deal of angst and anxiety about the implications of such an alert.

Participants who reflected on the cases and saw expected returns as an opportunity to learn were less likely to describe unmanageable anxiety and were even more accepting of situations where a trigger alert misfired (such as instances where a patient was intentionally discharged home overnight with a planned return for imaging in the morning).

Representative statements supporting each outcome are listed in Table 1.

**Table 1:** Representative statements supporting each outcome

Outcome	Representative Statement
Outcome # 1 Perceived increase in resource use	“[O]ccasionally when I ask or request that a patient be admitted to a service [it is because] I think that there may be a high likelihood that they would return for readmission, even if I didn't necessarily think on that day, they would be admitted I might advocate a lot more strongly for them to get admitted sooner. And I am not sure if that is a beneficial or detrimental thing to patient care and hospital resources” (Physician 5)
	“I think that we focus a lot on bounce-backs and that might actually create more inefficiencies in the emergency department because you are worried about those stats. Every good emergency doc should have a certain amount of bounce-backs otherwise, you are over consulting, and you are overworking other people which is very inefficient. So, I think indirectly the 72 hours can contribute to inefficiency” (Physician 1)
	“That was one where I was like oh interesting. I will get general surgery to admit more people with recurrent biliary colic. I don't know if that is a good thing or a bad thing, but it changed my practice” (Physician 3)
Outcome # 2 Elaboration of discharge instructions	“…the experience from the alert has taught me to spend a lot of time at the end with discharge instructions so that patients are aware of what to watch for and when to return… I don't necessarily mind getting 72-hour readmission alerts if I know that I discussed with the patient when they should return and then they did so based on those discussions. I think it has contributed to me giving broader more expansive discharge instructions” (Physician 5)
	“I put more detail in my discharges, especially in patients who I think may bounce-back. I think that that will help to clarify things for the subsequent physician” (Physician 4)
	“I think on the patients that I am more borderline on then I am more clear on their, like on the discharge summary that I am writing up with the concept that they are borderline and might require coming back and kind of what instructions are given to them about reasons to come back.” (Physician 13)
Outcome #3 Increased stress or anxiety	“…overall, these alerts and triggers create a lot of visceral gut reaction among staff…they can create a lot of anxiety for staff” (Physician 3)
	“…definitely more [anxiety] when I first started getting these and now as time has gone by, that level of panic when the email first comes has gone down substantially.” (Physician 6)
	“I felt like the 72-hour admission rates and the reasons for it weren’t held against me in any of my [performance] reviews …, it was more to help improve my practice and my performance. So, I didn't feel that it was penalizing in any way. I think that overall it helps me more than anything” (Physician 5)
	“… I also think people are just generally people feel anxious or feel worried or feel like they may be had a failure when they have bounced backs in general.” (Physician 17)
Outcome # 4 Review of individual cases	“… if I don't remember [the case] or if there was a discrepancy between my discharge diagnosis and the readmission then I go back, and I look them up and I figure out what happened. I guess reflect on my care and what happened and if there is anything that I can do to learn from it” (Physician 4)
	“If it is an unexpected then yes. I will pull the chart, review it in [the EMR] to start with. And look at labs and pull up the consultative report and hopefully eventually discharge summary.” (Physician 16)
Outcome #5 No change in behaviour	“[I] still practice the same way… it hasn't changed the amount that I consult, it doesn't change the amount that I investigate. I think for some individuals it might, just for the fear of having that statistic on their record.” (Physician 1)
	“In terms of it changing my practice, I don't think that any of the readmission alerts have ever changed my practice whatsoever.” (Physician 15)
	“[E]very so often it makes me think maybe I should have done something differently but overall I don't think that it has changed my practice significantly.” (Physician 10)

## Discussion

Our study suggests the variable impact of a 72-hour readmission A&F intervention. We identified a number of potential, often contradictory, outcomes associated with the trigger alert and the mechanisms and contexts that appear to contribute to them. Rather than simply relying on the assumption that an automated performance feedback strategy will work to change behaviour in a predictable way, implementers should realize the unpredictable nature of passive feedback delivery and consider implementing strategies to monitor and mitigate its variable effects.

Previous systematic reviews have demonstrated similar variability in A&F effectiveness on change in physician compliance with desired behaviour [1,14]. The studies contributing to such reviews approach feedback as the passive delivery of information using data report cards or one-on-one feedback but fail to consider the interactive and relational component of feedback that plays a role in determining how an individual will respond (and potentially change their behaviour). In contrast, Cooke et al. (2018) have previously explored the nature of physicians’ responses to receiving feedback and found that through audit and group feedback, a predictable cycle of six behaviours emerged [3]. They subsequently demonstrated that facilitated feedback could be used to help guide clinicians through the process of reacting to the data, understanding the data, and action planning in a peer group setting [15]. Recognizing the expected variability in outcomes, we are now offering facilitated feedback at our institution with the hope of improving the long-term outcomes of A&F interventions [15].

Participants universally noted anxiety related to trigger alerts. There was a clear difference between manageable anxiety that motivated physicians and contributed to their perception that this was a useful way to learn and the unmanageable anxiety that prompted people to prioritize behaviours that would limit the number of trigger alerts that they received. The relationship between anxiety and performance seems to respect findings first described by Yerkes and Dodson (1908) demonstrating that performance increases with physiological or mental arousal but only up to a point [16]. The model also suggests that when levels of arousal become too high, performance decreases [16]. Much like in simulation-based education, where simulation educators have embraced the potential benefits of adding stress with the expectation that it will improve learning outcomes, our data suggest that A&F implementers should be mindful that stress is a potential outcome of performance feedback [17]. When designing feedback strategies, administrators and the leadership should consider the outcome of stress and promote mechanisms that aim to generate manageable amounts of stress and minimize excessive amounts of stress. In particular, our results appear to suggest that the perceived linking of feedback to departmental performance review may have unintended downstream negative impacts on patients and health systems, a finding that should be considered carefully.

Rather than assuming that they can predict the effects of their A&F intervention, those implementing such interventions need to be aware of the potentially variable effects and consider implementing strategies to understand and monitor the downstream effects of their performance feedback strategies. Future studies should consider validating our study participants’ perceptions by developing measures to examine whether a 72-hour readmission alert actually results in increased resource usage, physician burnout, and/or comprehensive discharge documentation.

We acknowledge a number of limitations to this study. Some participants had received only a few alerts and the alert structure was changed shortly after our study was completed. This study should be seen as provisional in that some of the outcomes of the 72-hour readmission alert are yet to emerge and the acceptance that interventions will always mutate and thus can never be described fully [18].

More participants and a broader range of participants might have been engaged (a limitation primarily due to the required time budget and the willingness of individuals to participate). We nevertheless achieved theoretical saturation in our analysis and synthesis. It is possible that we were not able to recruit participants who had the greatest stress response to the alert (as these participants may have feared consequences to their participation) or only captured cohorts who had a strong enough response to reach out for further feedback. Overall, people seemed to feel comfortable voicing negative responses to the alert, and we are mindful that it would not be ethical or desirable to compel other individuals to participate.

Our approach of using qualitative methods gave us an appropriate lens with which to investigate mechanisms. “Mechanisms are embodied in the subjects’ reasoning, and they are best investigated therein… Program mechanisms change minds. They open eyes. And such close qualitative research is an ideal way of revealing such processes” [19]. Our use of these same methods to elaborate outcomes is more controversial. Realist evaluation works by explaining outcome patterns, and these cannot be determined through anecdotal remarks (on the part of subjects) or wishful thinking (on the part of evaluators) [19]. “Outcomes should be carefully conceptualized and indicators thought through; baselines should be established; before and after measures should be plotted; complete cohorts of subjects should be followed” [19]. To build on the work we have already done, it would be important to attempt to gather quantitative data that could further describe the outcome patterns proposed by our study. The key point is that in order for realist evaluation to be realized you need to have theory, qualitative evidence, and outcome data.

## Conclusions

The 72-hour readmission alert A&F strategy at our institutions generated a number of distinct outcome patterns resulting from a variety of mechanisms acting in different contexts. Our results suggest that the causal chain of performance feedback strategies is complex and non-linear. Existing studies in this area have not appreciated the role of human reasoning in the effects of A&F interventions. By developing generative explanations of causation, such as those sought in realist evaluation, we may gain insight into how to improve feedback programs that maximize desirable outcomes and minimize undesirable outcomes.

## Figures and Tables

**Figure 3:**
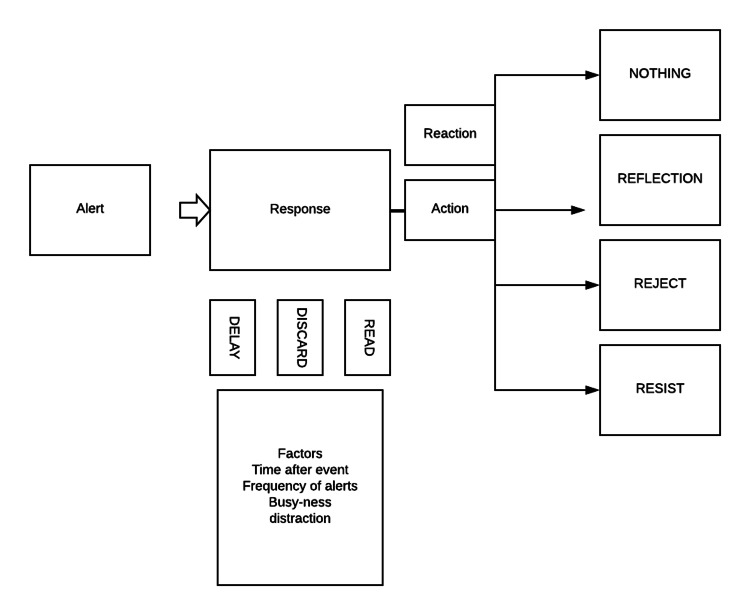
Initial program theory expressed as a logic model

## Appendices

Appendix A: Initial program theory and logic model

Appendix B: Guide for one-on-one semi-structured interviews regarding the 72-hour re-admission trigger alert

For your information, we will be recording this interview (are you ok with that)

Hello, my name is ______________ and I will be conducting semi-structured interviews on behalf of the study team to assess your perspectives of the 72-hour re-admission trigger alert notification. Through these discussions, we hope to understand the alert’s utility in fostering physician reflection and potentially improve the alert. This interview should take approximately 30-40 minutes of your time and you can choose to withdraw from the interview at any point.

As part of our commitment to confidentiality, we will be following several policies that protect those involved in the interview process. The original data will be kept in accordance with institutional policy. The transcripts made from the interview recordings will be anonymized and de-identified.

Thank you for donating your time to participate and shape the future of reflective feedback in emergency departments across the zone.

Initial impressions:

Participant interaction with the alert

1)    Do you receive trigger alert notifications?

2)    Do you always go to your institutional email and open and read the alert notifications?

a.     Why or Why not?

b.     Do you open the e-mail immediately or do you sometimes delay opening the e-mail? Why or why not?

c.     Have you ever discarded an alert without reading it?

d.     Are you aware that there is a survey question at the bottom of the alert email?

                                               i.     Have you ever completed the survey?

1.     Why or why not?

e.     Have you provided feedback to leadership (in ways other than the survey) regarding the trigger alert

Alert criteria and content

3)    Are you content with how frequently you receive the notifications? (do you find you get too many or do you think the criteria could be changed to get more ie. 96 hours instead of 72 hours) Why or why not?

4)    Are you content with the information provided in the notifications (Interviewer can show a sample alert to remind the participant of the information included in the alert)? Why or why not?

5)    Are you content with the criteria used to trigger the alert? Why or why not? 

72HourReadmissionRate Re-admission rate is calculated by comparing the discharge date of a visit with the first contact/triage date of the next subsequent ED visit. If the difference is less than72 hours AND the subsequent visit is also admitted, then visits will qualify for inclusion in this measure. Additionally, return visits are excluded when the return contact time is less than 18 hours from the index discharge time when the return visit presenting complaint is ‘imaging tests’ or when the diagnosis description of the index visit contains the term ‘private’ (means that they are returning for a particular consultant)

6)    If you could change the criteria used for the alert, how would you change them?

7)    Do you experience anxiety when you receive an alert?

a.     If so, do you think the amount of anxiety you experience is acceptable?

Re-admissions could be expected (ie. you’re not surprised the patient returned and was admitted) vs unexpected.

8)    Would you say the majority of 72-hour readmission alerts you’ve received are expected or unexpected?  Does your approach to using this feedback change depending on whether it was expected or not expected?

Utility of the alert

9)    Overall, do you find the alerts helpful? Why or why not?

a.     Do you believe that we should continue receiving the alerts? Why or why not?

10) Has the alert changed the way that you practice?

a.     If so, please explain how?

b.     If not, please explain why?

11) Do you find that you reflect more on the cases for which you receive a 72-hour alert compared to other cases (for which you have not received an alert)?

12) Do you feel that you have control over how you use the information from the 72-hour alert?

(some people have mentioned that they worry that the department is monitoring this metric and that physicians might be held accountable for their performance; do you feel this is the case or do you think you are free to independently determine how you use the information)

a.     Why or why not?

13) Do you find that the 72-hour alert gives you a greater understanding of “self”

a.     Makes you think beyond knowledge and skill problems and consider values and beliefs related to the patient (appendicitis example - physician felt the patient was malingering and sent home but ultimately had a true surgical emergency that was missed due to negative physician-patient interaction)

14) Do you find that the 72-hour alert improves your confidence in your ability to practice emergency medicine?

15) Have you used the feedback from the 72-hour alert to inform your practice?

a.     If yes, please explain

b.     If no, please explain why not

16) Have you used feedback from the 72-hour alerts to prompt you to acquire new knowledge or skills?

17) Have you used feedback from the 72-hour alert to change how you communicate with patients?

18) How does the 72-hr trigger alert email in our city compare with other reflective practice methods you have experienced during your training and practice?

Overall, do you have any thoughts about how the alert could be improved

Wrap-up: During this time the interviewer will quickly summarize the conversation held and will allow the participant to clarify or add information as needed.

Demographic information:

Gender:

Years in practice/year of training for residents:

Site of practice:

Thank you for taking the time to contribute to our study. We really appreciate your continued dedication to the faculty of Emergency Medicine in our city.

For the interviewer:

Please quickly summarize your thoughts on how the interview went, with emphasis on which questions brought forward interesting information or didn’t significantly contribute further to the content. Assess how your biases may have influenced the way in which you asked questions or recorded the answers

Appendix C

CMO Triads

Outcome #1: Perceived Increase in ED Resource Use

In the context where alert frequency rates are not widely available, the perception that alerts should be avoided promotes a perceived increase in resource use.

In contexts where the alert is used to make employment decisions, the perception that alerts should be avoided, uncertainty around the intention of the alert, and the perception that alerts reflect “mistakes” by the provider promotes a perceived increase in resource use.

In the context of early-career physicians, the perception that alerts should be avoided, uncertainty around the intention of the alert, and the perception that alerts reflect “mistakes” by the provider promotes a perceived increase in resource use.

In the context of minimal orientation to the basis and use for the 72-hour readmission alert, the perception that alerts should be avoided, uncertainty around the intention of the alert, and the perception that alerts reflect “mistakes” by the provider promotes a perceived increase in resource use.

In the context of the first few alerts received by a physician, the perception that alerts should be avoided, uncertainty around the intention of the alert, and the perception that alerts reflect “mistakes” by the provider promotes a perceived increase in resource use.

Outcome #2: Improved Discharge Communication (Written and Verbal)

In the context where the physician has a strong suspicion the patient may return to the ED, the belief that the physician is justifying their original discharge to colleagues or patients promotes a perceived improvement in discharge communication.

In the context where physicians have experience with receiving alerts, the belief that the physician is justifying their original discharge to colleagues or patients or the perception that physicians need to manage their anxiety around return visits promotes a perceived improvement in discharge communication.

In the context where physicians feel they are being monitored by department leadership, the belief that the physician is justifying their original discharge to colleagues or patients or the perception that physicians need to manage their anxiety around return visits promotes a perceived improvement in discharge communication.

In the context of competing demands on physicians during their clinical practice, the physician’s belief of a low likelihood of a patient returning with 72-hour readmission impedes discharge communication.

In the context of competing where the alert is seen as a nuisance, the physician’s belief that the management of a 72-hour return is straightforward or perceives that alerts are not valuable impedes discharge communication.

In the context of alerts firing inappropriately, the physician’s belief that the management of a 72-hour return is straightforward or perceives that alerts are not valuable impedes discharge communication.

Outcome #3: Increased Stress and Anxiety

In situations where multiple physician providers are involved in a patients’ care, the perception that the department is “tracking” alerts and using them against physicians promotes increased stress and anxiety.

In the context of limited leadership messaging regarding the alert purpose and use, the perception that the department is “tracking” alerts and using them against physicians, the belief that the alert frequency and content have an impact on hiring decisions and the perception that alerts reflect “mistakes” by the provider promote increased stress and anxiety.

In the context of early-career physicians, the perception that the department is “tracking” alerts and using them against physicians, the belief that the alert frequency and content have an impact on hiring decisions and the perception that alerts reflect “mistakes” by the provider promote increased stress and anxiety.

In the context of prior shame around alert-based cases, the perception that the department is “tracking” alerts and using them against physicians, the belief that the alert frequency and content have an impact on hiring decisions, and the perception that alerts reflect “mistakes” by the provider promote increased stress and anxiety.

In the context of the first few alerts received by a physician, the perception that the department is “tracking” alerts and using them against physicians, the belief that the alert frequency and content have an impact on hiring decisions and the perception that alerts reflect “mistakes” by the provider promote increased stress and anxiety.

In the context of a discrepancy between the index visit and return visit diagnosis, the perception that alerts reflect “mistakes” by the provider promotes increased stress and anxiety.

In the context where alerts and their frequency are addressed during performance reviews, the perception that alerts reflect “mistakes” by the provider promotes increased stress and anxiety.

In the context where alert frequency rates are not widely available, the perception that alerts reflect “mistakes” by the provider promotes increased stress and anxiety.

In the context where alert notifications arrive in the personal email, provider curiosity promotes increased stress and anxiety.

In the context where alert notifications arrive on vacations, weekends, or holidays, provider curiosity promotes increased stress and anxiety.

In the context where alert notifications are automated, provider curiosity promotes increased stress and anxiety.

In the context where providers receive alerts privately, the belief that alerts are a mechanism to improve and optimize care impedes increased stress and anxiety.

In the context where providers have access to other quality of care markers, the belief that alerts are a mechanism to improve and optimize care impedes increased stress and anxiety.

In the context where providers feel that they have control over the alert, the belief that alerts are a mechanism to improve and optimize care impedes increased stress and anxiety.

In the context where providers receive alerts privately, the belief that alerts are a mechanism to improve and optimize care impedes increased stress and anxiety.

In the context of provider job security, the belief that alerts are a mechanism to improve and optimize care impedes increased stress and anxiety.

In the context of a departmental culture that normalizes patient returns, the belief that alerts are a mechanism to improve and optimize care impedes increased stress and anxiety.

In the context of expected returns, the belief that alerts are not punitive impedes increased stress and anxiety.

In the context of providers with greater experience receiving alerts, the perception that alerts are not punitive and recognition that processing an alert requires thought and attention impede increased stress and anxiety.

Outcome #4: Review of the Case (Chart Review, Discussion With Other Physicians Involved)

In the context where the physicians feel personally responsible for the patient outcome, the perception that the alerts reflect “mistakes” by the provider and curiosity promotes a review of the case.

In the context of formal recognition from leadership that alerts are formative, curiosity promotes a review of the case.

In the context where physicians do not have other established approaches to patient follow-up, curiosity promotes a review of the case.

In the context of unexpected return visits, curiosity promotes a review of the case.

In the context of discordance between ED discharge and readmission diagnoses, curiosity promotes a review of the case.

In the context where there are competing demands on physicians during their clinical practice, a perception that the return was expected and there is little value to review impedes the review of the case.

In the context where the alert fires inappropriately, the perception that another physician or consultant was responsible for the patient or the perception that alerts are not valuable impedes the review of the case.

In the context where a physician feels that a return could not have been predicted, the perception that another physician or consultant was responsible for the patient or the perception that alerts are not valuable impedes the review of the case.
